# Supplementary material for: Adhirons are efficient tools to guide antiviral ligand discovery
Source: Commun Biol. 2025 Nov 26;8:1755. doi: 10.1038/s42003-025-09137-x (PMC12686443; doi:10.1038/s42003-025-09137-x)
Supplement: Supplementary file 1 — Supplementary Information [file 42003_2025_9137_MOESM1_ESM.pdf]

Supplemental material

AF1-01

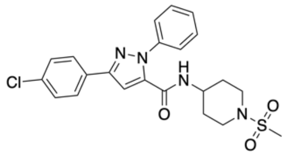

27395924

AF1-02

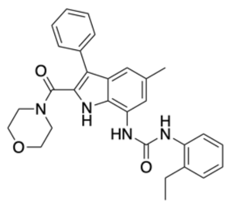

27225458

AF1-03

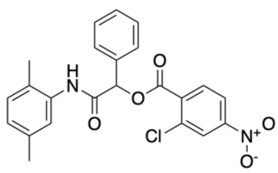

16656216

AF1-04

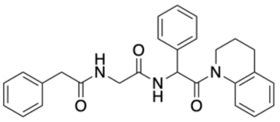

32750834

AF1-05

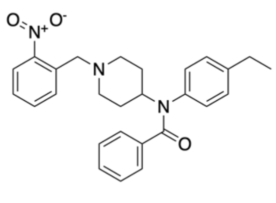

25393296

AF1-06

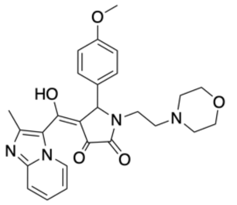

46531853

AF1-07

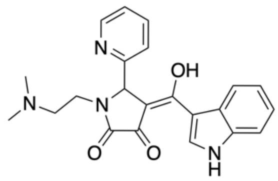

46512866

Supplementary Figure 1: *The AF1 compounds were generated as mimics of NP-Adhiron, an Adhiron inhibitor of CCHFV NP. For each compound the AF1 ID (top) and its eMolecules ID number (bottom) are shown.*

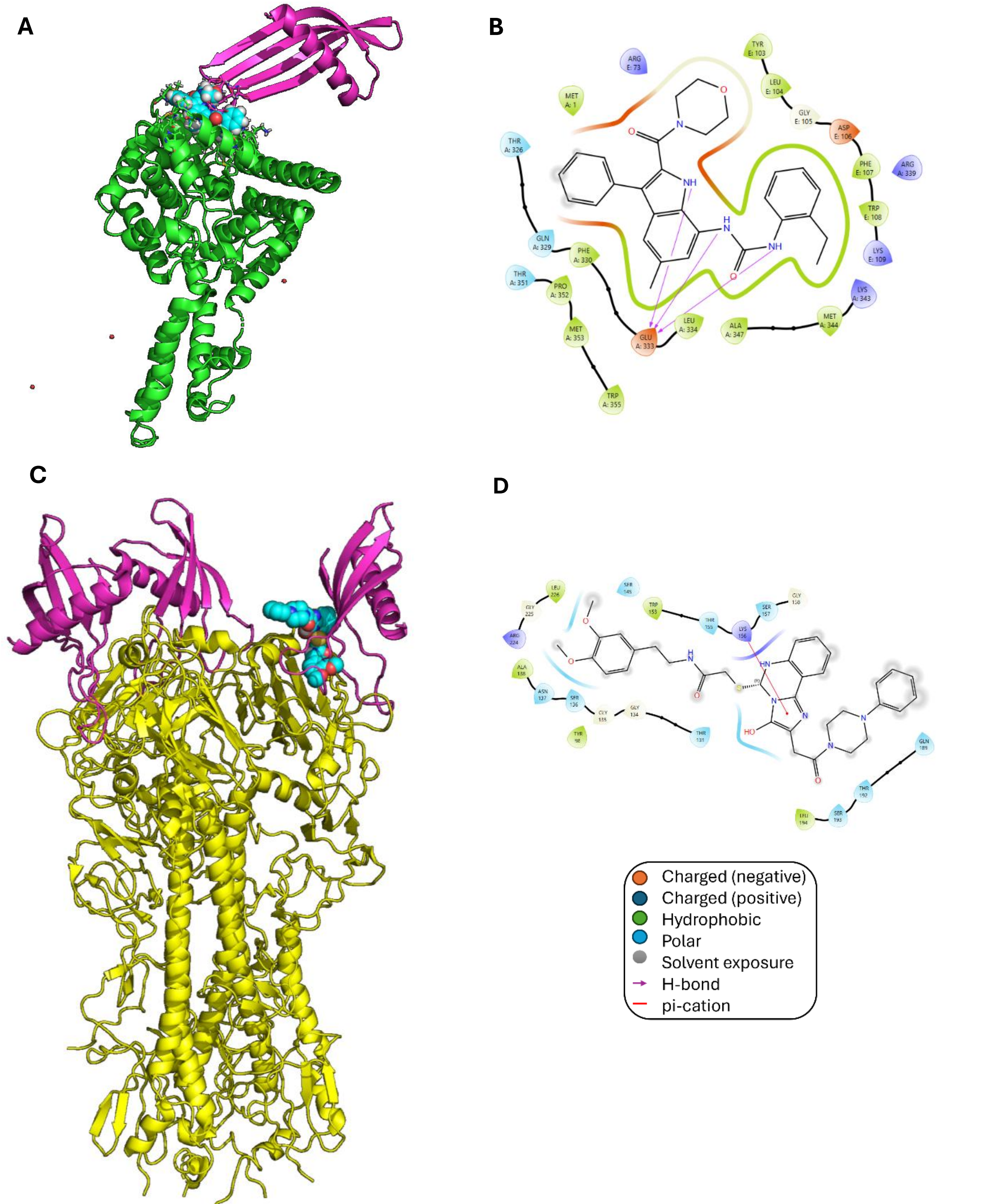

Supplementary Figure 2: Representative images of compounds from the AF and OA series docked to the Adhiron binding sites of their specific protein target. A) *AF compound bound to the Adhiron binding site. CCHFV protein shown as green ribbons, Adhiron shown as pink ribbons and AF compound as turquoise sphere.* ***B)*** *Ligand interaction diagram for AF series.* ***C)*** *OA compound bound to the Adhiron binding site in IAV HA. HA protein shown as yellow ribbons, Adhiron shown as pink ribbons and OA compound as turquoise sphere.* ***D)*** *Ligand interaction diagram for OA series.*

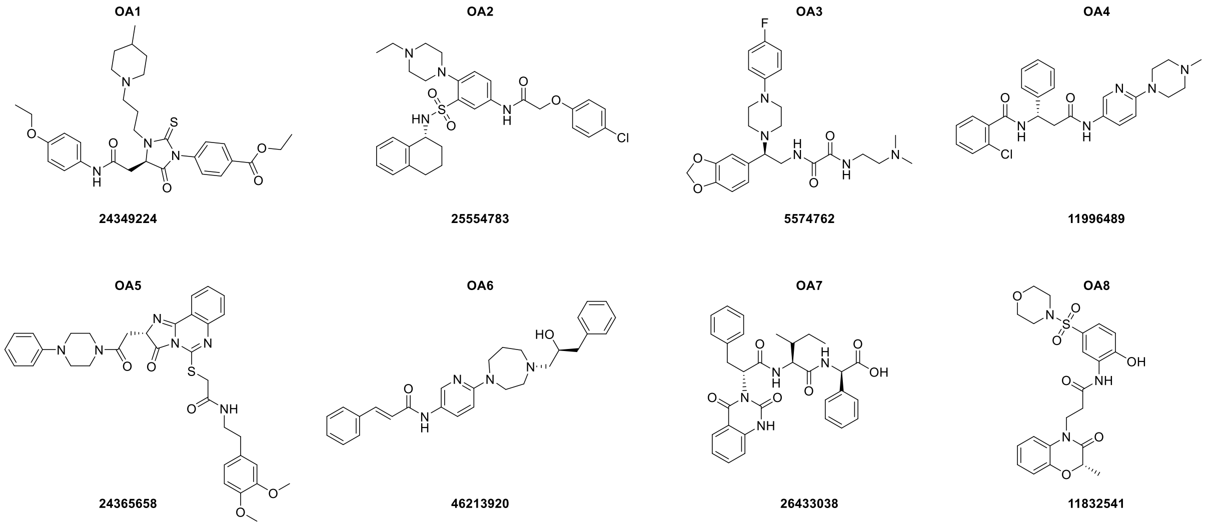

Supplementary Figure 3: *The OA compounds were generated as mimics of A5, an Adhiron inhibitor of IAV HA. For each compound the OA ID (top) and its eMolecules compound library ID number (bottom) are shown.*

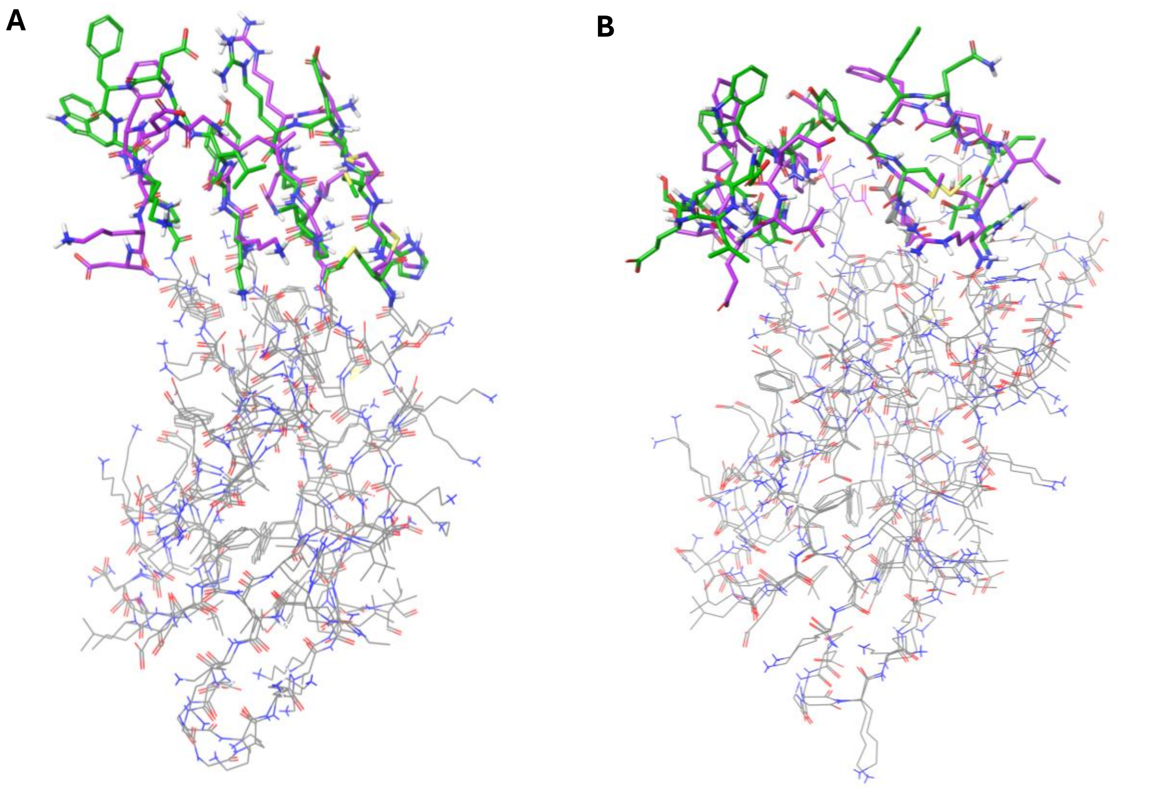

Supplementary Figure 4: Molecular dynamics simulation of CCHFV Adhiron (A) and A5 Adhiron (B) *Scaffold protein is shown as grey lines and loops shown as sticks. Green sticks show the first frame of the simulation and Purple the final frame of the simulations.*
