# Supplementary material for: Adhirons are efficient tools to guide antiviral ligand discovery
Source: Commun Biol. 2025 Nov 26;8:1755. doi: 10.1038/s42003-025-09137-x (PMC12686443; doi:10.1038/s42003-025-09137-x)
Supplement: Supplementary file 4 — Reporting Summary [file 42003_2025_9137_MOESM4_ESM.pdf]

nature portfolio

Corresponding author(s): Dr Katie Simmons

Last updated by author(s): 20/10/2025

Reporting Summary

Nature Portfolio wishes to improve the reproducibility of the work that we publish. This form provides structure for consistency and transparency in reporting. For further information on Nature Portfolio policies, see our Editorial Policies and the Editorial Policy Checklist.

Statistics

For all statistical analyses, confirm that the following items are present in the figure legend, table legend, main text, or Methods section.

n/a	Confirmed	
☐	☒	The exact sample size (*n*) for each experimental group/condition, given as a discrete number and unit of measurement
☐	☒	A statement on whether measurements were taken from distinct samples or whether the same sample was measured repeatedly
☐	☒	The statistical test(s) used AND whether they are one- or two-sided<br>*Only common tests should be described solely by name; describe more complex techniques in the Methods section.*
☒	☐	A description of all covariates tested
☒	☐	A description of any assumptions or corrections, such as tests of normality and adjustment for multiple comparisons
☐	☒	A full description of the statistical parameters including central tendency (e.g. means) or other basic estimates (e.g. regression coefficient) AND variation (e.g. standard deviation) or associated estimates of uncertainty (e.g. confidence intervals)
☐	☒	For null hypothesis testing, the test statistic (e.g. *F*, *t*, *r*) with confidence intervals, effect sizes, degrees of freedom and *P* value noted<br>*Give P values as exact values whenever suitable.*
☒	☐	For Bayesian analysis, information on the choice of priors and Markov chain Monte Carlo settings
☒	☐	For hierarchical and complex designs, identification of the appropriate level for tests and full reporting of outcomes
☒	☐	Estimates of effect sizes (e.g. Cohen's *d*, Pearson's *r*), indicating how they were calculated

Our web collection on statistics for biologists contains articles on many of the points above.

Software and code

Policy information about availability of computer code

Data collection	The 3D structure the Adhiron loop was used as a query molecule in the shape similarity search program ROCS 3.2.1.4<br>The compound library for screening was the eMolecules small molecule library, which had been converted to a maximum of 10 conformers/ compound using OMEGA 2.2.0.5<br>The shape similarity search program ROCS 3.2.1 was used to match commercially available small molecules to the adhiron loop structure.<br>Ligand docking was carried out using the Glide modules of Schrodinger (version 2023-4, Schrödinger, New York, NY, USA).<br>Molecular Dynamic Simulations were carried out using the Desmond module of Schrodinger (version 2023-4, Schrödinger, New York, NY, USA).
Data analysis	In order to identify the key Adhiron residues for mimic development, the Adhiron-bound CCHFV NP crystal structure (PDB: 6ZOO) was used as an input for the Protein Interfaces, Surfaces and Assemblies' service (PISA) hosted at the European Bioinformatics Institute.<br>Protein structures were visually inspected in PyMOL 2.3.2<br>The top ~150 scoring compounds from ROCS 3.2.1 were visually inspected using VIDA (OpenEye GUI).<br>A 2D chemical fingerprint was assigned to each compound using Canvas (Schrödinger Release 2025-1: Canvas, Schrödinger, LLC, New York, NY, 2025)<br>Ligand Docking and MD simulations were analysed using the Schrodinger GUI (version 2023-4, Schrödinger, New York, NY, USA).<br>Statistical Analysis using Microsoft Excel.

For manuscripts utilizing custom algorithms or software that are central to the research but not yet described in published literature, software must be made available to editors and reviewers. We strongly encourage code deposition in a community repository (e.g. GitHub). See the Nature Portfolio guidelines for submitting code & software for further information.

Data

Policy information about availability of data

All manuscripts must include a data availability statement. This statement should provide the following information, where applicable:

- Accession codes, unique identifiers, or web links for publicly available datasets
- A description of any restrictions on data availability
- For clinical datasets or third party data, please ensure that the statement adheres to our policy

All data supporting the findings of this study are available within the paper and its Supplementary Information.

Research involving human participants, their data, or biological material

Policy information about studies with human participants or human data. See also policy information about sex, gender (identity/presentation), and sexual orientation and race, ethnicity and racism.

Reporting on sex and gender	N/A
Reporting on race, ethnicity, or other socially relevant groupings	N/A
Population characteristics	N/A
Recruitment	N/A
Ethics oversight	N/A

Note that full information on the approval of the study protocol must also be provided in the manuscript.

Field-specific reporting

Please select the one below that is the best fit for your research. If you are not sure, read the appropriate sections before making your selection.

☒ Life sciences ☐ Behavioural & social sciences ☐ Ecological, evolutionary & environmental sciences

For a reference copy of the document with all sections, see nature.com/documents/nr-reporting-summary-flat.pdf

Life sciences study design

All studies must disclose on these points even when the disclosure is negative.

Sample size	For all biomolecular assays, at least three independent experimental replicates were performed (n=3) to get the same result in all replicates, as is standard practice in the field.
Data exclusions	No data was excluded.
Replication	For all biomolecular assays, at least three independent experimental replicates were performed (n=3) to get the same result in all replicates, as is standard practice in the field.
Randomization	Our data does not require randomization.
Blinding	No blinding experiments are included. An individual performed each experiment based upon expertise.

Reporting for specific materials, systems and methods

We require information from authors about some types of materials, experimental systems and methods used in many studies. Here, indicate whether each material, system or method listed is relevant to your study. If you are not sure if a list item applies to your research, read the appropriate section before selecting a response.

Materials & experimental systems

n/a	Involved in the study
☒	☐ Antibodies
☐	☒ Eukaryotic cell lines
☒	☐ Palaeontology and archaeology
☒	☐ Animals and other organisms
☒	☐ Clinical data
☒	☐ Dual use research of concern
☒	☐ Plants

Methods

n/a	Involved in the study
☒	☐ ChIP-seq
☒	☐ Flow cytometry
☒	☐ MRI-based neuroimaging

Eukaryotic cell lines

Policy information about cell lines and Sex and Gender in Research

Cell line source(s)	All cell lines were obtained from the European Collection of Cell Cultures (ECACC):<br>BHK 21 baby hamster kidney cells (85011433)<br>MDCK Madin-Darby canine kidney cells (85011435)
Authentication	Authentication was performed by ECACC and not in-house.
Mycoplasma contamination	Cells are routinely tested for mycoplasma contamination.
Commonly misidentified lines<br>(See ICLAC register)	No commonly misidentified cell lines were used in this study.

Plants

Seed stocks	N/A
Novel plant genotypes	N/A
Authentication	N/A
